# Malaria vectors diversity, insecticide resistance and transmission during the rainy season in peri-urban villages of south-western Burkina Faso

**DOI:** 10.1186/s12936-020-03554-5

**Published:** 2021-01-25

**Authors:** Dieudonné Diloma Soma, Serge Bèwadéyir Poda, Aristide Sawdetuo Hien, Moussa Namountougou, Ibrahim Sangaré, John Marie Emmanuel Sawadogo, Florence Fournet, Georges Anicet Ouédraogo, Abdoulaye Diabaté, Nicolas Moiroux, Roch Kounbobr Dabiré

**Affiliations:** 1 Institut de Recherche en Sciences de la Santé/Centre Muraz, Bobo-Dioulasso, Burkina Faso; 2 Université Nazi Boni, Bobo-Dioulasso, Burkina Faso; 3 Université Joseph Ki-Zerbo, Ouagadougou, Burkina Faso; 4 MIVEGEC, Univ. Montpellier, CNRS, IRD, Montpellier, France

**Keywords:** Vector, Bionomics, Resistance, Burkina Faso

## Abstract

**Background:**

This study reports an updated description on malaria vector diversity, behaviour, insecticide resistance and malaria transmission in the Diébougou and Dano peri-urban areas, Burkina Faso.

**Methods:**

Mosquitoes were caught monthly using CDC light traps and pyrethrum spray catches. Mosquitoes were identified using morphological taxonomic keys. PCR techniques were used to identify the species of the *Anopheles gambiae* complex and insecticide resistance mechanisms in a subset of *Anopheles* vectors. The *Plasmodium* sporozoite infection status and origins of blood meals of female mosquitoes were determined by ELISA methods. Larvae were collected, breed in the insectary and tested for phenotypic resistance against four insecticides using WHO bioassays.

**Results:**

This study contributed to update the entomological data in two peri-urban areas of Southwest Burkina Faso. *Anopheles* populations were mostly anthropophilic and endophilic in both areas and exhibit high susceptibility to an organophosphate insecticide. This offers an alternative for the control of these pyrethroid-resistant populations. These data might help the National Malaria Control Programme for decision-making about vector control planning and resistance management.

**Conclusions:**

This study contributed to update the entomological data in two peri-urban areas of Southwest Burkina Faso. *Anopheles* populations were mostly anthropophilic and endophilic in both areas and exhibit high susceptibility to an organophosphate insecticide. This offers an alternative for the control of these pyrethroid-resistant populations. These data might help the National Malaria Control Programme for decision-making about vector control planning and resistance management.

## Background

The World Health Organization (WHO) estimated to 229 million the number of cases of malaria and to 409,000 the number of death having occurred worldwide in 2019 [1]. The same year, 94% of all malaria deaths occurred in sub-Saharan African [1] countries, where malaria control consumes a major part of the national health budgets [2, 3].

The WHO’s global vector control strategy recommends the scaling up of long-lasting insecticidal nets (LLINs) and indoor residual spraying (IRS) to control malaria, towards achieving the Millennium Development Goals for malaria [4, 5]. Achieving high coverage of these interventions, especially to populations at highest risk of malaria, and their continued implementation remains a major challenge [6].

In Burkina Faso, malaria is endemic with an estimated number of annual cases reaching height million, resulting in 27,800 deaths [2]. Malaria control policies in Burkina Faso include intermittent preventive treatment (IPT) for pregnant women, Seasonal Malaria Chemoprevention (SMC) for children from 0 to 5 years old and the universal coverage with LLINs, according to the WHO recommendations [7–9]. In 2011 and 2012, the National Malaria Control Programmes (NMCP) of Burkina Faso in collaboration with President’s Malaria Initiative (PMI) implemented IRS as a pilot intervention in several villages of the Diébougou health district (South-West of Burkina Faso).

The implementation of insecticide-based vector control programs has led to the rapid emergence of physiological [10, 11] and behavioural [12–15] resistance mechanisms in many vector populations in Africa. In Burkina Faso, recent studies indicated that *Anopheles gambiae *sensu lato (s.l.) was highly resistant to both pyrethroids and organochlorine [16] insecticides, but showed low levels of resistance to carbamates and organophosphates [17]. Until now, no behavioural resistance mechanism (i.e. change in biting or resting behaviour) was clearly described in Burkina Faso in relation to the implementation of vector control measures. The spread of resistance mechanisms and changes in the vector population composition may lead to a reduced efficacy of the vector control interventions [11]. It is, therefore, essential to describe and monitor malaria vector bionomics, resistance, behaviour and contribution to malaria transmission in areas where vector control measures are implemented [18, 19].

Therefore, in order to gather relevant data to the NMCP for decision-making about vector control planning and resistance management, we monitored vector diversity, endophagy, resistance and malaria transmission during the 2015 rainy season in two peri-urban areas in Southwestern Burkina Faso. Both areas received universal pyrethroid LLIN distributions in 2010 and 2013 and one of both received bendiocarb IRS in 2011 and 2012 as part of a PMI pilot intervention.

## Methods

### Study areas

**Fig. 1:**
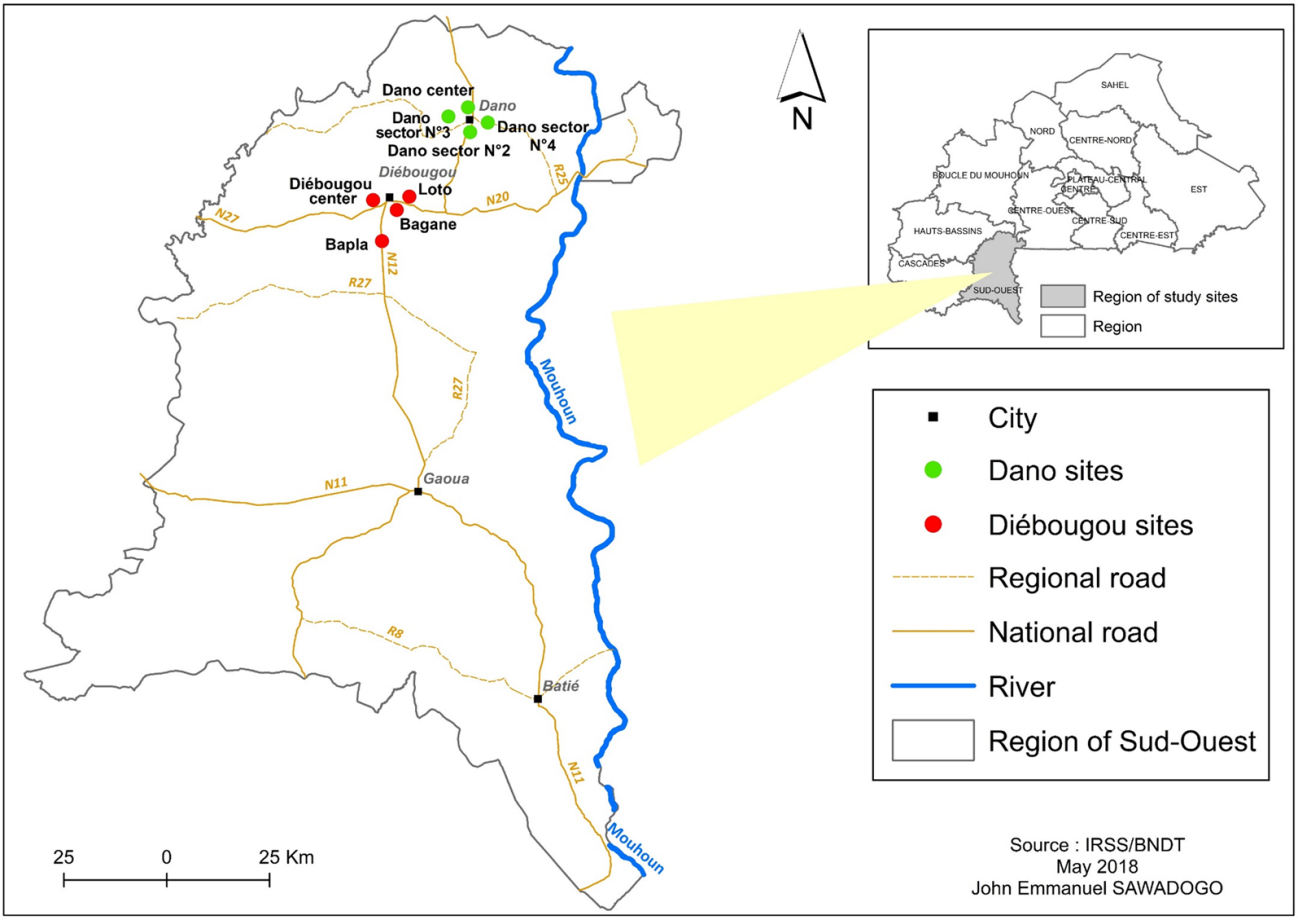
Location of the study areas and mosquito collection sites

The survey was carried out during the 2015 rainy season in the—29 km apart—peri-urban areas of Diébougou (N10.96741; W 003.24580) and Dano (N11.14288; W 003.05969) cities, in South-West Burkina Faso (Fig. 1). Both areas have similar environmental characteristics with an average 1000 mm annual rainfall occurring from May to October and a vegetation dominated by wooded savannah. Surveys were performed in height sites named Diébougou centre, Bagane, Loto and Bapla in the Diébougou area; and sector one to four in Dano. The main economic activity is agriculture in both areas where animals and humans use to live very closely in the same courtyard. In the Diébougou area, bendiocarb IRS was implemented in 2011 and 2012. Populations of both areas received free of charge LLINs in 2010 and 2013 in the framework of the NMCP national mass distribution.

### Study design and mosquito collections

Monthly mosquito collections were carried out from August to November 2015. One inhabited house was randomly selected in each site (i.e. 4 houses per study area). These houses were made of mud or cement with traditional roof or metal sheeting and were representative of the local housing. Mosquito collections were performed using CDC light trap both indoor and outdoor of each selected house during four successive nights from 18:00 to 06:00. Thus, 32 trap-night collections were performed per month and per area. Each month, early morning mosquito collections were also performed using pyrethrum spraying catches (PSC) inside 10 randomly selected inhabited houses over 4 consecutive days in each site (for a total of 40 houses per area per month).

### Laboratory processing of adult *Anopheles* (from CDC traps and pyrethrum spraying catches collections)

All collected *Anopheles* adults were morphologically identified under a stereomicroscope to the species complex and preserved in 1.5 ml tubes containing silicagel [20]. Unfed females form CDC light traps belonging to the *An. gambiae* complex were dissected to determined their physiological age using the Detinova method [21]. A randomly selected sub-sample (25 female per house per month) of females collected in light traps and belonging to the *An. gambiae* complex were proceed by Polymerase Chain Reaction (PCR) for species identification following the protocol described by Santolamazza et al. [22]. Female infection by *Plasmodium falciparum* was also assessed on the same subsample using enzyme-linked immunosorbent assays (ELISA) technique described by Wirtz et al. [23]. Abdomen of blood-fed females collected indoor by PSC were analysed for identification of blood meal source by the ELISA technique [24].

### Bioassay

*Anopheles* larvae were collected from natural breeding sites throughout the two peri-urban areas of Diébougou and Dano. In each study area, mosquito larvae were collected in at least 10 breeding sites distant from each other by at least 200 m. Larvae were then pooled together, brought back to the IRSS insectarium and reared under controlled conditions (temperature 27 ± 2 °C, Relative humidity 70 ± 10%) until adult’s emergence. Non-blood-fed 3–5 days-old females morphologically identified as *An. gambiae* s.l. were put in contact with DDT 4%, deltamethrin 0.05%, bendiocarb 0.1% and chlorpyriphos methyl 0.1% impregnated filter papers following the WHO standard protocol [25]. Four replicates of 20–25 individuals were exposed to each tested insecticide. *Anopheles gambiae “Kisumu”* strain was used as the susceptible control strain [25]. Mortality was recorded 24 hours after exposure. PCR analyses were then conducted on a subsample of 200 females per area to detect the *kdr* (L1014F) and *ace-1* (G119S) mutations using the protocol described by Martinez-Torres et al. [26] and Weill et al. [27], respectively.

### Entomological indicators and statistical analysis

Mosquito density per trap per night was calculated for each malaria vector species as the number of *Anopheles* individuals collected per trap per night. Endophagy rate (ER) was the proportion of mosquito collected indoors from CDC light traps. Parous rate (PR) was calculated as the proportion of parous *An. gambiae *s.l. among dissected individuals. The *Plasmodium*- sporozoite rate (SR) of infection was calculated as the proportion of mosquitoes positive for CSP-ELISA. Human Blood Index (HBI) was the proportion of mosquitoes found to be fed on Humans relative to the total number tested.

All other statistical analyses were performed using the software R version 3.6.0 [28]. A generalized linear mixed model (GLMM) fitting a negative binomial distribution of the error was applied to compare *Anopheles* densities between areas and species. The collection site, position (indoor or outdoor) and date were included in the model as random intercepts (sites and positions were nested). The human blood index of both *An. gambiae *s.l. and *Anopheles funestus* were compared between areas using logistic regression. The SR, PR, and ER were compared between areas using binomial GLMMs fitted on individual data and for both *An. gambiae* s.l. and *An. funestus*. The collection site and date were included in the model as random intercepts. GLMM were fitted using the glmmTMB function of the glmmTMB package [29]. The post-hoc Tukey’s method was used to perform multiple comparison among modalities of the fixed terms. The ‘emmeans’ function of the ‘emmeans’ package [30] was used to compute Density rate ratios (DRR) and Odds ratios (OR) with 95% confidence intervals for Negative Binomial and Binomial models, respectively. The allelic frequencies of the *kdr* and *ace*-1 mutations in *An. gambiae* s.l. populations were calculated and compared using the ‘GenePop’ package in R [31].

## Results

### Malaria vector morphological identification, densities and behaviour

A total of 9 625 *Anopheles* mosquitoes was collected (6006 by CDC traps and 3619 by PSC) (Table 1). *Anopheles gambiae* s.l. was the most abundant species in both areas whatever the collection method (84.3% by CDC traps and 92.7% by PSC), followed by *An. funestus* group (13.2% by CDC traps and 6.7% by PSC) (Table 1). Only 24 (0.4%) *Anopheles nili* individuals were caught, all by CDC traps (Table 1).

**Table 1:** *Anopheles* densities in the study areas collected in 2015

Districts	Sampling method	*An. gambiae* s.l.		*An. funestus* group		*An. nili*		Total
		n	%	n	%	n	%	
Diébougou	CDC	1862	*73.30*	664	*26.14*	14	*0.55*	2540
	PSC	1394	*88.00*	190	*11.99*	0	*0.00*	1584
Dano	CDC	3202	*92.38*	254	*7.32*	10	*0.28*	3466
	PSC	1962	*96.41*	73	*3.58*	0	*0.00*	2035

*PSC* pyrethrum spray catches, *CDC* CDC light traps, *n* number of mosquitoes

**Fig. 2:**
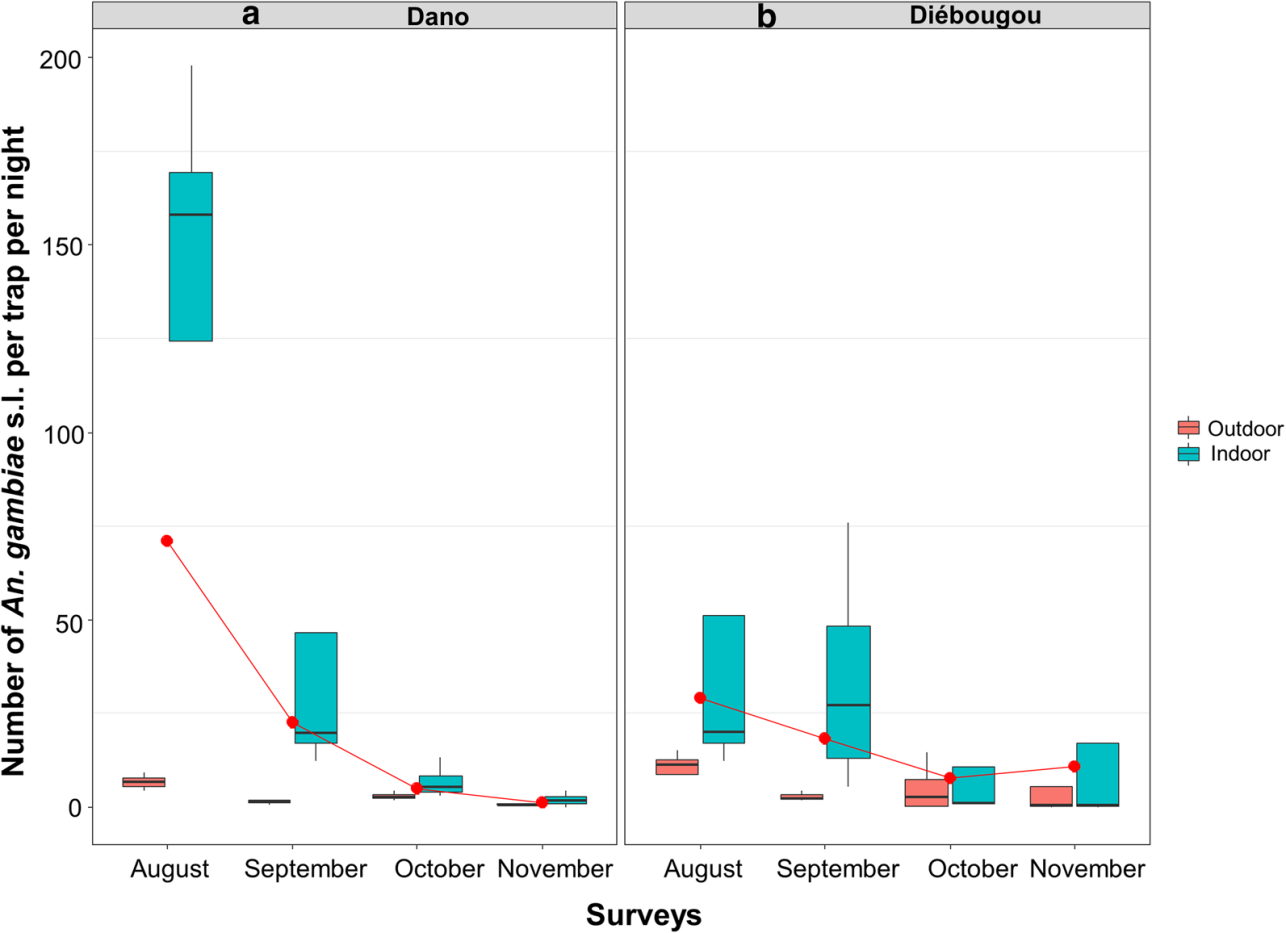
Mean nightly densities of *An. gambiae *s.l. collected using light-traps. Boxes indicate inter-quartile range (IQR) and median of number of *An. gambiae* s.l per trap. The upper whisker extends to the largest value no further than 1.5* IQR from the hinge. Red dots show the mean nightly densities per month

**Fig. 3:**
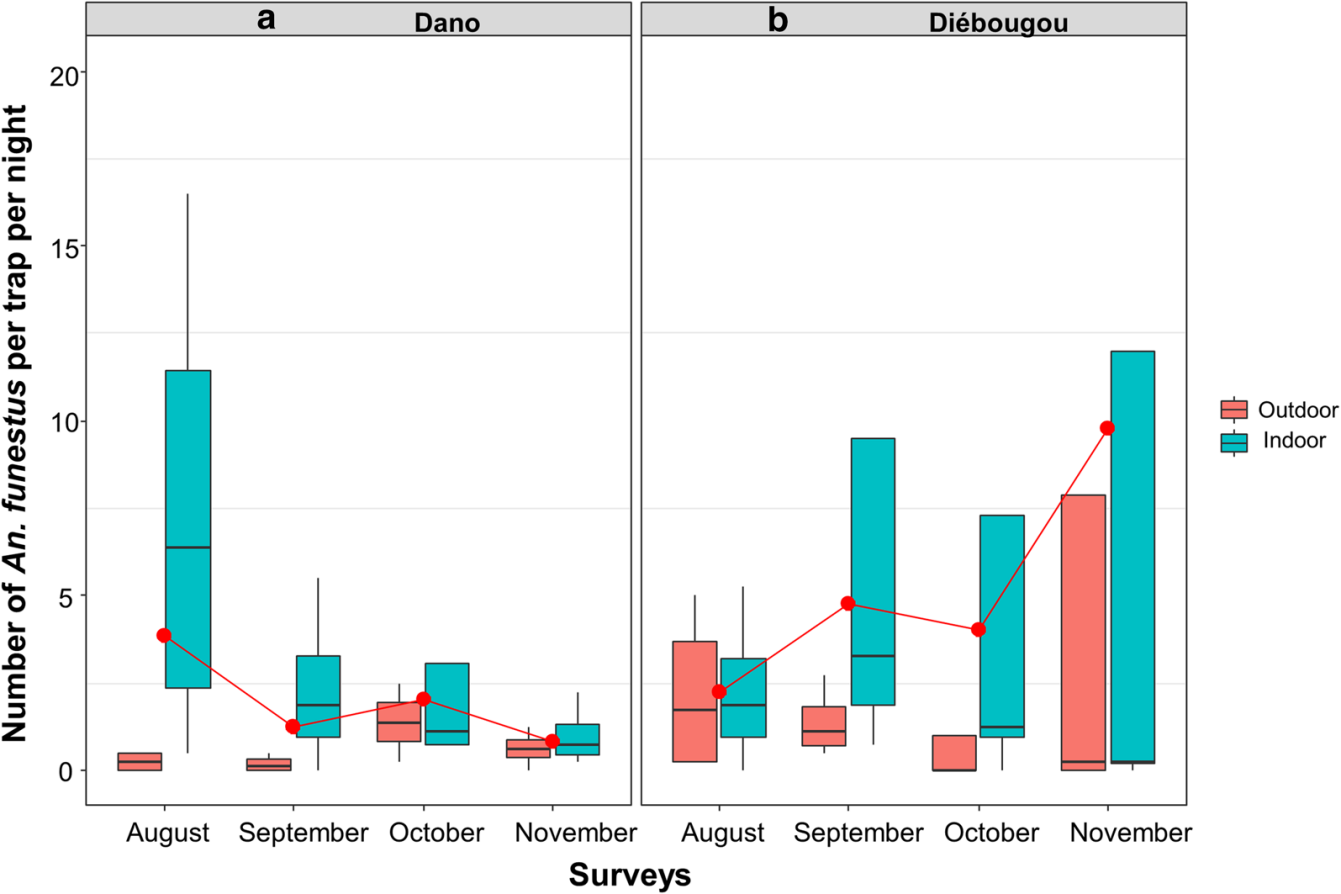
Mean nightly densities of *An. funestus* group collected using light-traps. Boxes indicate inter-quartile range (IQR) and median of number of *An. funestus* group per trap. The upper whisker extends to the largest value no further than 1.5* IQR from the hinge. Red dots show the mean nightly densities per month

Regarding mosquitoes collected by CDC light trap, the mean density of *Anopheles* per trap and per night was 21.9 in Diébougou not significantly different than 27.1 in Dano (Density Rate Ratio DRR = 1.10, IC95% [0.23; 5.17]; P = 0.89). The mean density of *An. gambiae* s.l. per trap and per night was 25.0 in Dano, higher than 16.6 in Diébougou (DRR = 1.25, IC95% [0.27; 5.83]; P = 0.77). Densities were at their maximum during August in both area (71.1 and 29.1 in Dano and Diébougou, respectively) and decreased the following months (Fig. 2a, b). The mean density of *An. funestus* group per trap per night was 5.1 in Diébougou, higher than 1.9 in Dano (DRR = 0.80, IC95% [0.17; 3.79]; P = 0.77). In Dano, *An. funestus* densities decreased from 3.8 to 0.8 between August and November while during the same period in Diébougou, they increased from 2.2 to 9.7 (Fig. 3a, b).

**Fig. 4:**
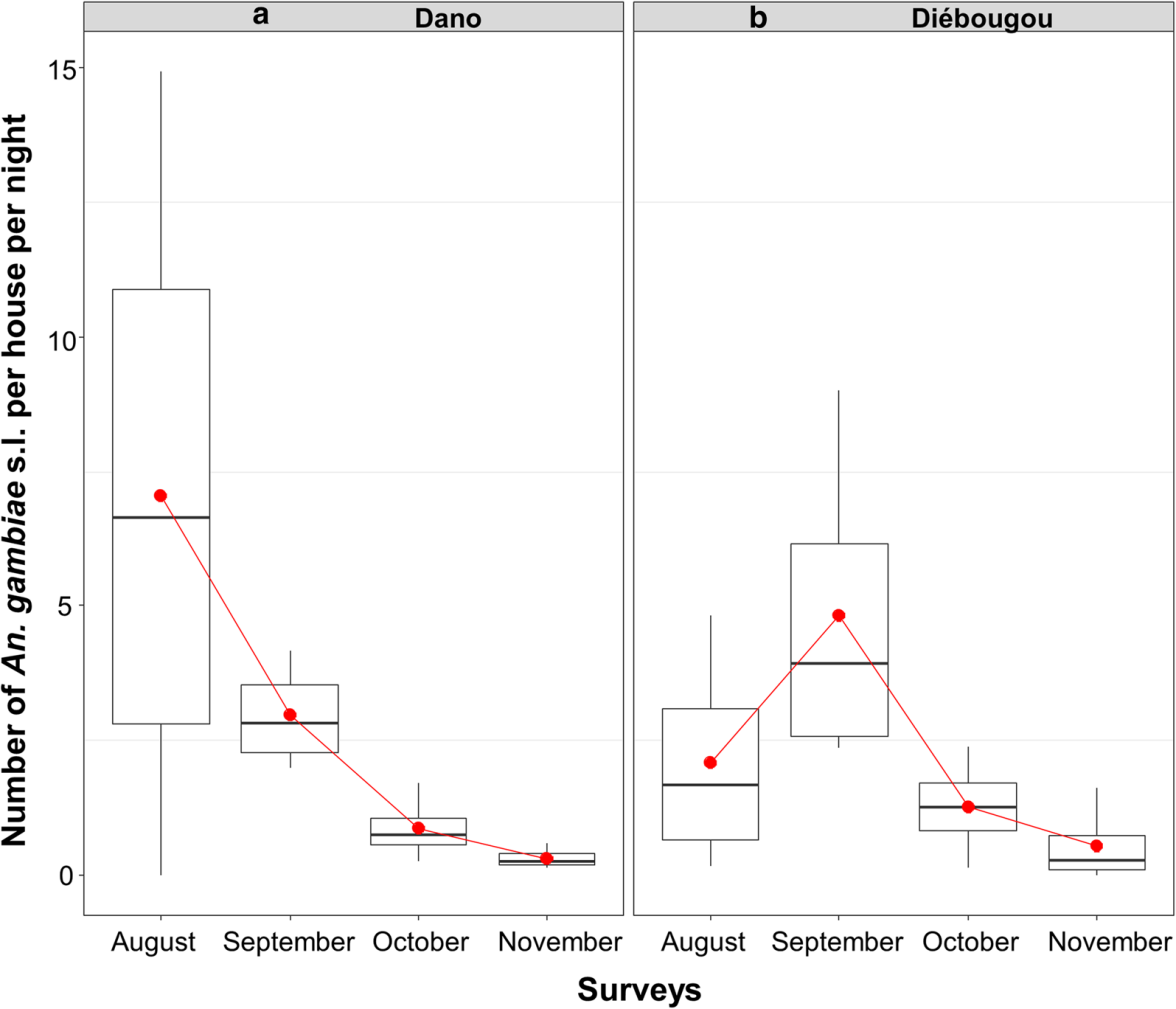
Mean indoor resting densities of *An. gambiae *s.l. Boxes indicate inter-quartile range (IQR) and median of number of *An. gambiae *s.l. per house. The upper whisker extends from the hinge to the largest value no further than 1.5* IQR from the hinge. Red dots show the mean indoor resting densities per month

**Fig. 5:**
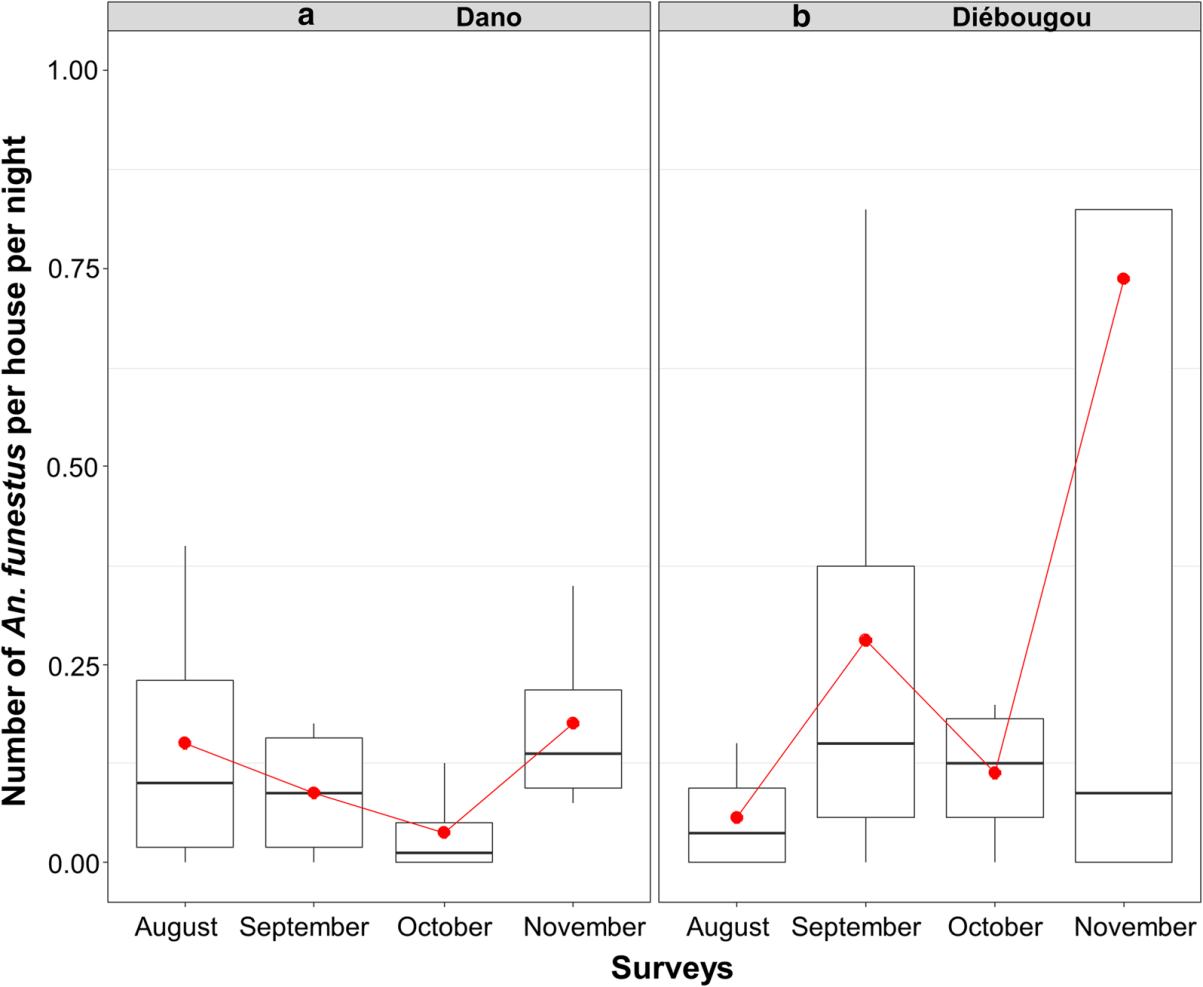
Mean indoor resting densities of *An. funestus group*. Boxes indicate inter-quartile range (IQR) and median of number of *An. funestus* group per house. The upper whisker extends from the hinge to the largest value no further than 1.5* IQR from the hinge. Red dots show the mean indoor resting densities per month

The mean density of *Anopheles* per house collected by PSC was 2.90 in Dano and 2.47 in Diébougou (Density Rate Ratio DRR = 0.92, IC95% [0.37; 2.25]; P = 0.85). Indoor resting densities of *An. gambiae *s.l. were the highest in August in Dano (7.0) than in September in Diébougou (4.8; Fig. 4a, b). Indoor resting densities of *An. funestus* group were the highest in November in both areas (0.17 and 0.73 in Dano and Diébougou respectively, Fig. 5a, b).

Mean Endophagy rate (ER) of *An. gambiae *s.l. as estimated by the GLMM was 89.1% [95%CI] [67.2; 97.0] in Dano significantly higher than 70.0% % [36.9; 90.3] in Diébougou (OR [95% CI] 3.50 [1.08; 11.3], P = 0.03). Mean Endophagy rate (ER) of *An. funestus* group was 83.8% [67.8; 92.7] in Dano not significantly different than 67.8% [45.5; 84.2] in Diébougou (OR [95% CI] 2.46 [0.90, 6.69], P = 0.07).

### Molecular identification of *Anopheles gambiae*

Over the 400 *An. gambiae *s.l. individuals selected per area for molecular identification, 84.50% (338/400) from Diébougou and 95.50% (382/400) from Dano were successfully identified. *Anopheles gambiae *sensu stricto (s.s.) was the most represented species belonging to the *An. gambiae* complex in both study areas (70.5%, n = 338 in Diébougou and 68.2%, n = 382 in Dano) followed by *Anopheles coluzzii* (22.1%, n = 106 in Diébougou and 23.5%, n = 132 in Dano) (Table 2) and *Anopheles arabiensis* (7.3%, n = 35 in Diébougou and 8.2%, n = 46 in Dano).

**Table 2:** Species composition of *Anopheles gambiae* complex 2015

Districts	*An. gambiae* s.l.	*An. gambiae* s.s.		*An. coluzzii*		*An. arabiensis*	
		n	%	n	%	n	%
Diébougou	479	338	*70.5*	106	*22.1*	35	*7.3*
Dano	560	382	*68.2*	132	*23.5*	46	*8.2*

*n* number of mosquitoes

### *Plasmodium* infection

The mean sporozoite rate [IC95%] of *An. gambiae *s.l. as estimated by the GLMM was 7.7% [2.7–19.9] in Dano and 13.2 [5.1–30.6] % in Diébougou. The SR of *An. gambiae *s.l. did not differ significantly between areas (OR = 0.55; IC95% [0.17; 1.72]; P = 0.29).

*An. funestus* group sporozoite rate was estimated to be 4.6% [2.1–9.6] in Dano and 1.4% [0.01–3.1] % in Diébougou. The mean SR of *An. funestus* group in Dano was significantly higher than that in Diébougou (OR = 3.29; IC95% [1.09; 9.96]; P = 0.03).

### Parous rate and blood-feeding preference in *Anopheles gambiae* s.l.

The mean parous rate [IC95%] as estimated by the GLMM was 66.25% [60.96–71.17] in Dano, not significantly different than in Diébougou (64.44% [58.56–69.91]; OR = 1.25; [0.88, 1.79]; P = 0.21). A total of 1552 blood-fed *Anopheles* (626 from Dano and 926 form Diébougou) were tested for blood feeding preference (human, cattle, donkey, pig) (Table 3). Overall, the proportion of *An. gambiae* s.l. that fed on human was 68.1% (408/599) in Dano and 68.8% (516/749) in Diébougou. The overall human blood index did not differ significantly between Dano and Diébougou (OR = 0.81; IC95% [0.47; 1.38]; P = 0.22). The proportion of *An. funestus* group females that fed only on human was 51.8% (14/27) in Dano, not significantly different than 61.0% (108/177) in Diébougou (OR = 0.65; IC95% [0.11, 3.77]; P = 0.4).

**Table 3:** Blood meal source of *An. gambiae* s.l. and *An. funestus* group from Diébougou and Dano areas in 2015

Sites/species	Animals					Human		Mixed		Total
	Cattle	Donkey	Pig	Other	%	n	%	n	%	n
Diébougou										
*An. gambiae *s.l.	35	2	1	176	28.5	516	68.8	19	2.5	749
*An. funestus* group	3	0	1	51	31.0	108	61.0	14	7.9	177
Total	38	2	2	227	29.0	624	67.3	33	3.5	926
Dano										
*An. gambiae *s.l.	4	1	1	127	22.2	408	68.1	58	9.6	599
*An. funestus* group	1	0	0	9	37.0	14	51.8	3	11.1	27
Total	5	1	1	136	22.8	422	67.4	61	9.7	626

*n* number of blood-fed *Anopheles* females, *Other* other animals not determined, *Mixed* fed on both animal and human

### *Anopheles gambiae* susceptibility to insecticides

**Fig. 6:**
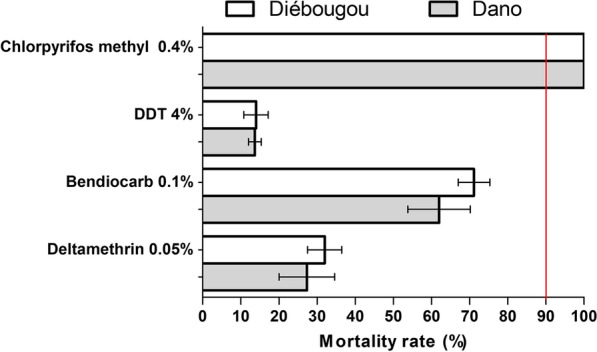
Mortality rates recorded in WHO cone test of wild populations of *An. gambiae* s.l. originated from Diébougou and Dano exposed to four insecticides. *Red vertical line indicates the resistance threshold according to the WHO. 95% confidence intervals are shown*

Mortality rates of *Anopheles gambiae *s.l. from both areas were 100% with chlorpyrifos methyl 0.4% (Fig. 6) indicating full susceptibility to this insecticide. With benthiocarb 0.1%, mortality rates were 71.1% and 62.0% in Diébougou and Dano, respectively, indicating resistance. With deltamethrin 0.05%, mortality rates were 32% and 27.33% in Diébougou and Dano, respectively. When exposed to DDT 4%, mortality rates were only 14% and 13.6% in Diébougou and Dano, respectively (Fig. 6).

### Allele frequencies of the *kdr* and *ace-1* mutations

The frequencies of the *kdr* mutation were high in both *An. coluzzii* (0.93 and 0.74 in Diébougou and Dano, respectively) and *An. gambiae sensu stricto* (s.s.) (0.84 and 0.85 in Diébougou and Dano, respectively) (Table 4). For *Anopheles arabiensis*, this mutation was detected only in individuals from Diébougou and at a moderate frequency (0.342) (Table 4). Frequencies of *kdr* mutation differ significantly between areas (Diébougou and Dano) in *An. arabiensis* (exact G test P < 0.0001 and *An. coluzzii* (exact G test P < 0.001) but not in *An. gambiae *s.s. (exact G test P = 0.53).

**Table 4:** Allelic and genotypic frequencies at the *kdr-west* and *ace-1* locus in *An. gambiae* s.l. populations from Diébougou and Dano areas

Species	Sites	n	Genotypes *kdr*			f(L1014F)	Genotypes *ace 1*			f(119S)
			1014L	1014L	1014F		119G	119G	119S	
			1014L	1014F	1014F		119G	119S	119S	
*An. arabiensis*	Diébougou	19	12	1	6	0.342	19	0	0	0.000
	Dano	21	21	0	0	0.000	21	0	0	0.000
*An. coluzzii*	Diébougou	94	6	0	88	0.936	87	7	0	0.037
	Dano	98	12	27	59	0.740	89	9	0	0.046
*An. gambiae *s.s	Diébougou	238	36	3	199	0.842	223	15	0	0.032
	Dano	231	21	23	187	0.859	220	11	0	0.024

*N* number of mosquitoes, *f(1014F)* frequency of the 1014F resistant *kdr* allele, *f(119S)* frequency of the 119S resistant *ace-1* allele

The results showed very low frequencies of the *ace-1* mutation in all areas and species (0.037 and 0.046 for *An. coluzzii* in Diébougou and Dano, respectively; 0.032 and 0.024 for *An. gambiae *s.s. in Diébougou and Dano, respectively; Table 4). These frequencies did not vary between areas (*An. coluzzii* : exact G test P = 0.79; *An. gambiae *s.s.: exact G test P = 0.55). We failed to detect any homozygous resistant (RR) individual for this mutation (Table 4).

## Discussion

The entomological monitoring that we carried out revealed that *An. gambiae *s.l. and *An. funestus* group were the main malaria vectors in both Diébougou and Dano areas. The density of *An. gambiae *s.l. (major vector) fell down drastically in October and November, compared to the two previous months. This was also true for *An. funestus* in Dano. However, in Diébougou, we collected more *An. funestus* individuals in October and November (especially in November). The predominance of *An. gambiae *s.l. could be explained by the presence of its preferential deposits, consisting in temporary shallow and sunny water collections associated with rainfall [32, 33]. The increased densities of *An. funestus* group at the end of the rainy seasons in Diébougou might be explained by the presence, in the Bapla site, of a dam that provides permanent and semi-permanent breeding sites typically associated with the presence of this species [33–36]. Similar observations have been reported by Dabiré et al. [37] in two savannah villages (Soumousso and Lena) in Burkina Faso, where *An. funestus* group was found as the major malaria vector towards the end of the rainy season.

Mean parous and sporozoite infection rates of *An. gambiae* s.l. were high, indicating that older females were more prevalent and probably capable of malaria transmission during the rainy season in both study sites. These results corroborate previous studies carried out in the savannah areas of Bobo-Dioulasso, Burkina Faso [38] and Gansé, Côte d’Ivoire [39]. These data highlight the need for the correct use of protective tools such as LLINs. This situation could contribute to reduce human-vector contact and induce a decrease in the human population that is not very infectious for the vectors as well as mortality in the epidemiologically dangerous stages (parous female) [40, 41].

Despite many years of continuous implementation of indoor, insecticide-based, vector control measures (LLINs alone or in combination with carbamate-IRS) in the study areas, *Anopheles* populations continue to exhibits mainly anthropophilic and endophilic behaviours, similarly to prior descriptions in closed area [37]. This seems to indicate, at the opposite to what was found in other areas [12, 18, 19], that LLINs and IRS did not induced significant change in vectors behaviour in Dano and Diébougou. This situation may be explained by the high pyrethroid-resistance levels [42, 43] observed in the vector population well before the 2010 and 2013 LLINs distributions.

In both areas of this study, *An. gambiae* populations were resistant to bendiobarb, DDT and pyrethroid. The intensive use of these insecticides in agriculture (gardening, rice and cotton growing) as well as in public health (IRS, LLINs) was found to induce selection of insecticide resistance in malaria vectors [44]. Cotton is intensively cultivated around Dano and Diébougou and was shown to possibly induce strong selection pressure malaria vectors [45]. This constitutes a limit to the efficacy of vector control strategies based on pyrethroids (such as LLINs) and carbamates in this part of the country. However, susceptibility tests indicated that *An. gambiae* was still susceptible to chlorpyriphos-methyl, an organophosphate that received a recommendation for its use in IRS [46]. This insecticide family might therefore be used in this area of Burkina Faso in combination with pyrethroids-LLIN with the goal to manage pyrethroids resistance and help reduce malaria transmission [47, 48].

In this study, we characterize phenotypic resistance of *An. gambiae *s.l. and identify the *kdr* mutation as a probable major cause for pyrethroids and DDT resistance. However, we did not investigated the role of metabolic resistance (such as esterase, oxidase and glutathione-*S*-transferase) that might have contributed to pyrethroids resistance and explained carbamate resistance [49, 50]. Moreover, insecticide resistance in *An. funestus* was not investigated. Because this species is a major malaria vector in the area, particularly at the end of the rainy season, further studies should consider describing phenotypic resistance and the mechanisms involved.

## Conclusions

This study contributed to update the entomological data in two peri-urban areas of Southwest Burkina Faso. *Anopheles* populations (*An. gambiae *s.l. and *An. funestus* group) were mostly anthropophilic and endophilic in both areas. Furthermore, the high susceptibility of vector populations to organophosphates offers an alternative for the control of these pyrethroid-resistant populations. These data might help NMCP for decision-making about vector control planning and resistance management. However, it is necessary to characterize insecticide resistance in the *An. funestus* population in order to get the whole picture.

## Data Availability

The datasets used during the current study are available from the corresponding author on reasonable request.

## Abbreviations

WHO: World Health Organization
CDC: Center Disease Control
LLINs: Long-lasting insecticidal nets
IRS: Indoor residual spraying
IPT: Intermittent preventive treatment
SMC: Seasonal malaria chemoprevention
NMCP: National Malaria Control Programmes
PMI: U.S. President’s Malaria Initiative
PSC: pyrethrum spraying catches
PCR: Polymerase chain reaction
ELISA: Enzyme-linked immunosorbent assays
